# Gynecological and Obstetrical Society of Baltimore

**Published:** 1893-02

**Authors:** William S. Gardner

**Affiliations:** Secretary


### GYNECOLOGICAL AND OBSTETRICAL SOCIETY OF BALTIMORE.

### NOVEMBER MEETING.

The President, DR. B. B. BROWNE, in the chair.

DR. GEORGE H. ROHE read a paper entitled "Gynecological Work Among the Insane."

The subject was treated under three heads:

1. Is it necessary?
2. Is it practicable?
3. What are the results?

To show the necessity of the work, it was stated that of thirty-five insane women examined, twenty-six, or 74.3 per cent. showed some evidence of pelvic disease or abnormality. The lesions found were mostly tears of the perineum or cervix, uterine displacements with adhesions, adhesions of the tubes and ovaries, cystic ovaries, parovarian cysts, etc.

DR. ROHE expressed the belief that a careful examination by a competent gynecologist would show that at least fifty per cent. of all insane women had some form of pelvic disease. This large percentage of diseased pelvic organs among the insane certainly indicated the necessity for gynecological treatment among this class of patients.

That this work can be successfully carried out among insane women is evidenced by the report of eighteen cases of abnormal section with removal of the tubes and ovaries. The patients were affected with the various clinical forms of mental disturbance: melancholia, mania, periodic mania, hysterical mania, puerperal insanity and hystero-epilepsy.

The duration of the insanity in the various cases was from one to eleven years. In all but one case the insanity had lasted over a year, and in very few were there any good prospects of recovery under the usual management.

At the date of reading the paper, three of those operated upon had been discharged recovered, and in ten there had been decided improvement in physical and mental symptoms. Two had died after the operation, one from sepsis, and one in *status epilepticus.*

These results are believed by Dr. Rohe to justify the prosecution of gynecological work among the insane. The plea is made that the insane woman is as much entitled to relief from physical ills as is her sane sister. No argument beyond the recital of the facts should be necessary to enforce this view.

DR. WILLIAM P. CHUNN: I have operated upon two cases of hystero-epilepsy—in both I removed the ovaries. Both recovered from the operation and both were improved in general health. I would be very loath to operate upon any case where examination failed to discover to the touch appreciable disease. It is well to differentiate between epilepsy and hysteria. The former is, in my opinion, incurable. If a woman is made pevish, iracible and melancholy, or unreasonable from pain, or the dread of pain, she may be called nervous or hysterical, but this is not epilepsy.

Personally, I would not take out ovaries or tubes for hysteria or epilepsy unless disease could be demonstrated before or at the time of operation.

DR. WILMER BRINTON : I have seen three cases of puerperal insanity in private practice. They were treated for a while at home, but they became so violent the families were compelled to send them to an institution for the insane. All three cases died there, and their deaths occurred within ninety days after delivery. I believe that the greater number of cases of puerperal insanity result from septic inoculation at the time of delivery, or from some lesions of the genital tract.

DR. THOMAS OPIE : Dr. Chunn has remarked upon the fact that in several of Professor Rohe's cases, there was no pathological state of the ovaries discovered, and that therefore the operation for their removal was contra-indicated.

There are occasionally met with cases where our manual explorations reveal no physical change in these organs, and yet their functioning is disordered in the most positive way.

The following case will serve to illustrate my point :

M. R. ———, age thirty-one, single. Menstruation began at thirteen, and continued regularly and without pain until sometime between sixteen and eighteen years of age, when she first manifested a condition of delirium at her monthly periods.

Her abnormal menstruation was accompanied by still more pronounced disturbance of this function and greater mental alienation in the way of inability to concentrate her thoughts and a confusion of ideas. When at the age of twenty-eight, there was observed at her periods a twitching of the lower limbs, a short time before the appearance of her flow. This lasted during its continuation and for a week afterwards.

With each recurrence, these attacks increased in severity, gradually involving the upper extremities in clonic spasms. A year prior to operation she began to lose consciousness during the attacks. Occasionally, between her periods she had muscular twitching, which always passed off in a few hours. Until this time, the invalid had been able to attend to light duties in connection with her home.

For eight months prior to operation she was totally disqualified for all duties—mental and physical—indeed was bedridden.

Upon admission to the Hospital, May 18th, she was in a most debilitated and anemic state physically, associated with well marked mental aberration. Her look was confused, there was momentary stupor, she became convulsed, her head was thrown back, muscles were rigid, pupils contracted, skin moist, urinary secretions scanty, respiration increased, temperature normal. When the attack was over, she stated on being interrogated, that she remembered nothing about the attack.

An oophoriotomy was performed May 21st. The ovaries were found to be relatively small and perfectly normal. June 9th, no recurrence of spasms, her mind clear and active. June 27th, patient has entirely recovered from operation and has no neurotic symptoms. Five months have elapsed since the removal of the ovaries. She is restored mentally and physically to a state of health.

There was no neurotic family history in this case.

Her troubles began coincidently with her earliest menstruation. At first they stimulated the aura. The attacks accompanied her ovulation for eighteen years—growing year by year worse, until she was upon the brink of ruin, both as to mind and body.

The ovaries on removal gave all the appearances of being normal.

DR. J. WHITRIDGE WILLIAMS: I have listened with interest to the conservative views expressed by Dr. Ashby, regarding the removal of the uterine appendages for psychical disorders, and wish to add my voice to his in condeming the indiscriminate castration of women under all sorts of pretexts. Unless we are able to diagnose a pathological lesion of the tubes and ovaries, we should not think of attempting to remove them, either for the relief of pain or as an experimental measure in psychical disorders. For it is only when they present a marked pathological lesion that we can be at all sure of our results after operation, and we are liable to be accused of reckless surgery if we remove apparently normal organs purely for the cure of some evil, of whose cause we are yet in ignorance.

Recent work on the nerves of the ovary gives us more of a basis upon which to base the doctrine of reflex ovarian disturbances, for vön Herff has shown that the ovary contains nerves which supply all parts of the organ. These are so abundant that he states that they not only are present in large quantities, but that they may be said to compose a considerable portion of the bulk of the ovary.

WILLIAM S. GARDNER.
Secretary.

### VAGINAL CAPSULES.

During the past year I have made an extensive use of Anderson's vaginal capsules in the treatment of various forms of uterine and vaginal disease.

Formerly, I was obliged to make applications of absorbent cotton through the speculum, which required personal attention on my part, every day or two. The use of these capsules (by means of which we secure the prolonged action of any desired medicament) has proved a saving of time to myself and expense to those who could ill afford it. They are especially serviceable where the physician cannot for any reason, see his patient sufficiently often, and she can readily prepare and introduce them herself. In short, the Anderson capsule is cleanly, convenient and effective.

Yours truly,

ARCH. LYBOLT, M. D.

New York City.

